# Paradoxical sex differences in a hamster model of angiotensin II-dependent hypertension and associated renal injury

**DOI:** 10.1186/s13293-025-00755-y

**Published:** 2025-11-03

**Authors:** Hong Ji, Laura German do Nascimento, Jungeun Ahn, Dong Hyang Kwon, Gabrielle Williams, Xie Wu, Robert C. Speth, Seth A. Hawks, Nisha K. Duggal, Juan M. Saavedra, Kathryn Sandberg, Aline M. A. de Souza

**Affiliations:** 1 Department of Medicine, Division of Nephrology and Hypertension, Georgetown University, Washington, DC USA; 2 Department of Pathology, Georgetown University, Washington, DC USA; 3 Department of Pharmacology & Physiology, Georgetown University, Washington, DC USA; 4 Department of Pharmaceutical Sciences, Berry and Judy Silverman College of Pharmacy, Nova Southeastern, Fort Louderdale, FL USA; 5 Department of Biomedical Sciences and Pathobiology, Virginia Polytechnic Institute and State University, Blacksburg, VA USA

**Keywords:** Angiotensin II, Hypertension, Sex differences, Syrian hamster, Kidney, Inflammation, Renin-angiotensin system, IL-6, ACE2, AT1

## Abstract

**Background:**

Biological sex is a critical determinant in cardiovascular and renal disease outcomes. Although angiotensin II (Ang II) infusion is widely used to model hypertension in mice and rats, little is known about its effects in the Syrian hamster, a small rodent increasingly used for translational research. This study aimed to develop a model of chronic Ang II-induced hypertension in Syrian hamsters and investigate sex-specific differences in blood pressure, renal pathology, and components of the renin-angiotensin system (RAS).

**Methods:**

Male and female Syrian hamsters (8–9 weeks old) were infused subcutaneously with Ang II (200 ng/kg/min) or saline via osmotic minipumps for four weeks. Mean arterial pressure (MAP) and kidney wet weight were determined on the euthanasia day. The kidneys were analyzed for renal pathology; renal RAS enzymes (ACE and ACE2) were measured by colorimetric assay and qPCR; cytokines (IL-6 and IL-1β) were measured by qPCR; and the angiotensin receptor type 1 (AT1R) was measured by radioligand binding and qPCR.

**Results:**

Ang II infusion increased MAP in both sexes but elicited a significantly greater response in females (+ 50 mmHg) than males (+ 27 mmHg, *p* < 0.005). Female hamsters exhibited pronounced kidney injury, including acute tubular necrosis, glomerular sclerosis, and vascular fibrinoid necrosis, along with a 2-fold increase in kidney weight normalized to body weight. Ang II significantly downregulated renal ACE, ACE2, and AT_1_R expression and activity in females but not in males. Renal IL-6 and IL-1β mRNA levels were elevated 20-fold and 3.9-fold, respectively, in females, compared to modest increases in males.

**Conclusions:**

Female Syrian hamsters exhibit heightened vulnerability to Ang II-induced hypertension and renal damage compared to males, marked by exaggerated blood pressure elevation, enhanced renal inflammation, and suppression of classical RAS components. This novel hamster model provides a unique platform for studying sex-specific mechanisms of hypertension and renal pathology, with translational relevance for subpopulations of women who are at increased risk of Ang II-dependent hypertension-associated renal disease.

## Abstract (plain language summary)

High blood pressure (also known as hypertension) is a major risk factor for heart and kidney disease. Men and women often experience these conditions differently, but scientists still don’t fully understand why. In this study, researchers focused on how male and female Syrian hamsters respond to a substance called angiotensin II, which is known to raise blood pressure and is commonly used in research to mimic high blood pressure in animals.

Over four weeks, male and female hamsters were given either angiotensin II or a harmless salt solution. The researchers measured blood pressure and looked at changes in kidney health and in a system of hormones that helps control blood pressure, called the renin-angiotensin system. They found that angiotensin II caused a much bigger rise in blood pressure in female hamsters than in males. The female hamsters also showed more severe kidney damage, including scarring and inflammation. Important hormones and enzymes that usually help regulate blood pressure were found to be lower in females after treatment, while markers of inflammation were much higher.

These results suggest that female hamsters are more sensitive to angiotensin II and may be more at risk of developing severe kidney problems from high blood pressure. This new hamster model offers a valuable way to study how men and women differ in their response to high blood pressure and could help researchers find better, more personalized treatments—especially for women who may be more vulnerable to certain types of hypertension-related kidney disease.

## Highlights

- Developed a novel model of angiotensin II-induced hypertension in Syrian hamsters.
- Female hamsters showed significantly higher increases in blood pressure than males in response to angiotensin II.
- Females exhibited more severe kidney damage, including tubular necrosis, glomerular sclerosis, and vascular injury.
- Only in females, angiotensin II reduced key components of the renal renin-angiotensin system (ACE, ACE2, AT1R).
- Inflammatory markers (IL-6, IL-1β) were dramatically elevated in female kidneys compared to males.

## Introduction

In 1941, Parker and Bradley [1] reported that intravenous administration of angiotonin (now known as angiotensin II, Ang II) increased blood pressure in men. Angiotensin II administration was also shown to increase blood pressure in other mammalian species, including mice [2], rats [3], cats [4], dogs [5], pigs [6], sheep [7], and non-human primates [8]. Consequently, Ang II infusion has become a widely used animal model of hypertension, with mice and rats being the most common species studied. As of June 2025, Pubmed Lists over 5500 papers on the role of Ang II in blood pressure regulation in the rat and over 4000 in mice, in contrast to studies in dogs (640), sheep (520), cats (220), pigs (290) and non-human primates [48].

Rats and mice are preferred animal models for biomedical research for a variety of reasons, which includes the ease and cost-effectiveness of housing small rodents compared to larger animals. Hamsters are also a small rodent belonging to the subfamily Cricetinae, of which the Syrian hamster has been the most studied. Until now, hamsters have not been explored as a model of Ang II-dependent hypertension. Here we establish a chronic model of Ang II-dependent hypertension in the hamster and investigate the impact of biological sex in this new model.

Ang II-dependent hypertension is associated with chronic kidney disease and renal inflammatory disease [9]. Inhibitors of Ang II synthesis and blockers of the Ang II type 1 receptor (AT_1_R), which mediates the vasoconstrictor effects of Ang II, are widely prescribed clinically to reduce kidney disease progression [10]. Thus, many experimental studies of hypertension have focused on the impact of blood pressure on the mechanisms of kidney pathology and renal inflammation [11].

The majority of studies using hamsters to model disease have focused on mechanisms of cardiomyopathy involving the renin angiotensin system (RAS), including the etiology of vascular dysfunction and role in heart failure [12]. Less is known about the hamster kidney in models of chronic renal disease or about the renin angiotensin system (RAS) in the hamster kidney. Therefore, we investigated the impact of exogenously administered Ang II on the metabolism and catabolism of Ang II in the kidney by measuring the mRNA expression and enzyme activity of renal angiotensin converting enzymes (ACE) and ACE2. Ang II mediates its vasoconstrictor effects in part through the angiotensin type 1 receptor (AT_1_R) that is highly expressed in the glomeruli and renal tubules in the renal cortex. Thus, we measured the mRNA expression in the renal cortex and radioligand binding of the AT_1_R in glomeruli. In addition, the effects of Ang II infusion on renal pathology and renal cytokine expression were assessed.

## Methods

### Hamsters

Female and male Syrian hamsters obtained from Envigo (Frederick, MD; now a division of Inotiv as of 2021) were acclimated in a temperature-controlled animal facility in the Division of Comparative Medicine at Georgetown University for 3–5 days prior to use. Hamsters were ~ 8–9 weeks of age at the time of procedures. All animal experiments complied with USDA guidelines, Animal Research: Reporting of in vivo experiments (ARRIVE), and were approved by the Georgetown University Institutional Animal Care and Use Committee (IACUC), protocol 2020-0063. Same sex animals were housed two per cage and were given Laboratory Rodent Diet (LabDiet, Richmond Indiana) and tap water *ad libitum* under controlled conditions (12 h light/dark schedule at 24 °C). Body weight (BW), food, and water intake were monitored twice weekly.

### Angiotensin II (Ang II) infusion

Mini-osmotic pumps (Alzet model 2004) were aseptically filled with human angiotensin II (Millipore-Sigma A9525 or Phoenix Peptides 002–12) dissolved in sterile saline to release 200 ng/kg/minute, or sterile saline for the vehicle control. The hamsters were anesthetized with isoflurane, and pumps were implanted subcutaneously for 4 weeks.

### Mean arterial blood pressure (MAP) and heart rate (HR)

On the last day of the Ang II infusion, hamsters were anesthetized with isoflurane (3–5% induction and 1–3% maintenance at 1 L/min oxygen) and placed on a heated table to maintain body temperature at 37 °C. A polyethylene catheter connected to a pressure transducer (MLT0699; ADI Instruments, Bella Vista, Australia) and a signal amplifier (ETH-400; CB Sciences Inc., Milford, MA, USA) was implanted in the femoral artery. The analog signal from the amplifier was digitized using a 12-bit analog-to-digital converter (PowerLab/400; ADI Instruments). The pulsatile arterial pressure was recorded at 1000 Hz using Windows software (Chart v. 7.0, ADI Instruments). Pulse to pulse analysis was used to calculate MAP and HR from the pulsatile arterial pressure measurements. After baseline measures were determined, values for MAP and HR were obtained by averaging the values over a 10 min-period [13].

### Renal cortex isolation

The kidneys were removed within 5 min at the time of euthanasia surgery under isoflurane anesthesia. For mRNA analyses, total RNA was immediately extracted from isolated renal cortex using TRIzol reagent (Life Technologies, Carlsbad, CA). For enzyme activity and AT_1_R radioligand binding, isolated renal cortex tissue was finely chopped, placed into 1.5 mL Eppendorf tubes, and flash frozen in liquid nitrogen and maintained at −80 °C until enzyme activity and AT_1_R binding were determined.

### Angiotensin-converting enzyme (ACE), angiotensin-converting enzyme-2 (ACE2), AT_1_R and cytokine mRNA expression

Total RNA from renal cortex was extracted using TRIzol reagent (Life Technologies, Carlsbad, CA). First strand cDNA was made from total RNA using iScript cDNA synthesis kit (BioRad, Hercules, CA) with MMLV RNase H^+^ reverse transcriptase, oligo(dT), and random hexamers. Quantitation of specific mRNAs and GAPDH (for control) were performed by real-time PCR using the QuanStudio 3 qPCR System (Thermo Fisher Scientific, Waltham, MA). The PCR reaction mixture consisted of RNase free water, PowerUp™ SYBR™ Green Master Mix (Thermo Fisher Scientific), 300 nM specific primers (Table 1) and cDNA samples. PCR reactions without reverse transcription were included to control for contamination by genomic DNA. The tissue expression of these genes was calculated using the 2 - ΔΔ CT method, which enables relative quantitation (treated sample is X fold of control sample) through measurements of crossing thresholds (CT).

**Table 1:** Primer sequence

Primer name & Reference	Forward	Reverse
ACE [53]	AGGAGATGGTGGGCTCAGAT	TTGGCTTCTGCGTACTCGTT
ACE2 [54]	GAAGAGGCTGTCAGGTTGTC	TGCCAACCACTACAATTCCC
AT_1_R [53]	ACAGCTATGGAATACCGCTGG	GCCACCAGCATTGTGCGTC
GAPDH [55]	GTGGAGCCAAGAGGGTCATC	GGTTCACACCCATCACAAACAT
IL-6 [56]	GGACAATGACTATGTGTTGTTAGAA	AGGCAAATTTCCCAATTGTATCCAG
IL-1b [57]	CGGCAGGTGGTGTCAGTCAT	GGAGCATCAGCCACGATCAG

### ACE and ACE2 enzyme assays

Renal cortex samples (approximately 50 mg protein/sample) were rapidly thawed and homogenized in 6 vol/tissue of ice-cold lysis buffer, which included the following protease inhibitors: 0.50 µg/mL pepstatin A, 0.25 µg/mL leupeptin, 0.1 mg/mL bacitracin, and 0.57 mmol phenylmethylsulfonyl fluoride. Protein concentration for each sample was determined by a NanoDrop spectrophotometer with absorbance recorded at 280 nm. Enzyme activity of ACE and ACE2 were measured using fluorogenic assays with internally quenched fluorogenic substrates Abz-Phe-Arg-Lys(Dnp)-Pro-OH (Enzo, BML-P161-0001) and Mca-Ala-Pro-Lys(Dnp)-OH (Enzo, BML-P163-001), respectively. Enzymatic assay reactions were performed using 96-well microtiter plates in a total volume of 100 µL: 80 µL Reaction Buffer (1 M NaCl, 0.1 mM ZnCl_2_, 75 mM Tris, pH 7.5), 10 µL of sample, and 10 µL of substrate. The amount of renal cortex protein used was 10 µg and 20 µg of renal cortex homogenates for the ACE and ACE2 assays, respectively. Substrate concentration curves revealed that non-substrate Limiting conditions were reached at 100 µM and 60 µM substrate for ACE and ACE2, respectively (*data not shown*). The formation of the product was determined at 37 °C by following the increase in fluorescence over time using a fluorescence plate reader at an excitation wavelength of 320 nm and an emission wavelength of 410 nm. The initial velocities were determined from the rate of fluorescence increase over a 0–180-minute period for ACE and ACE2 activity, and by analyzing the period corresponding to the pseudolinear range. For ACE activity, total peptidase enzyme activity was measured in the presence of a vehicle (reaction buffer), and for ACE2 in the presence of captopril. ACE activity was calculated as total peptidase activity minus nonspecific ACE activity, which was peptidase activity in the presence of the ACE inhibitor, captopril (30 µM). Specific ACE2 activity was determined as non-ACE activity minus nonspecific ACE2 activity, which was total peptidase activity in the presence of captopril (30 µM) and the ACE2 inhibitor MLN-4760 (30 µM).

### Angiotensin II type 1 receptor (AT_1_R radioligand binding)

Forty mg of renal cortex was rapidly thawed and homogenized using a polytron in an ice-cold 20 mM hypotonic sodium phosphate buffer (pH 7.2). Homogenates were centrifuged to precipitate the cell membranes at 42,000 x g for 20 min at 4 °C. An assay medium buffer (AM5, pH 7.2), composed of 150 mM NaCl, 5 mM disodium EDTA, 0.1 mM bacitracin, and 50 mM dibasic sodium phosphate, pH 7.2 was used to resuspend the cell membranes, which were rehomogenized then recentrifuged as above and finally resuspended in the AM5 buffer (20 mg initial weight per mL). Membrane protein was measured by Nanodrop (Thermo Fisher). Membrane protein (40 µg in 50 µL) was incubated for 90 min at approximately 22 °C in a volume of 100 µL with ^125^I-[Sar^1^,IIe^8^]-Ang II (prepared at Georgetown University as described previously [14] at six concentrations ranging from 0.2 to 4 nM as described previously [15]. Nonspecific binding was determined in the presence of the AT_1_R antagonist losartan (10 µM). Binding reactions were terminated by rapid filtration through a Brandel cell harvester. Filter-bound ^125^I was counted in a Cobra II gamma counter. *Quantitation*: Specific AT_1_R binding was defined as the total amount of radioligand bound minus nonspecific binding (defined as the amount bound in the presence of 10 µM losartan). Data points were obtained in duplicate. K_d_ and B_max_ values were determined using the one-site nonlinear regression analysis program in PRISM (GraphPad Software, Inc).

### Histology

The kidneys were collected and kept in formalin for 24 h and transferred to 70% ethanol. The tissue was paraffin-embedded, sectioned, and stained at the Histopathology & Tissue Shared Resource at Georgetown University. Six kidneys per group were examined independently by two pathologists (D. Kwon and J. Ahn) blinded to each sample group assignment. Semi-quantitation of the degree of acute tubular injury and acute tubular necrosis (ATI & ATN) and Tamms-Horsfall Protein was performed with the following grading scale: 0–4 [0 (normal);1, affected area up to 1–25% (minimal); 2, affected area 26 − 50% (moderate); 3, affected area 51–75% (moderate-severe); and 4, affected area 76 − 100% (severe)]. The presence of other pathologic findings, including glomerular sclerosis, glomerular congestion, glomerular fibrinoid necrosis/ischemic change, interstitial inflammation, tubular calcification, and vascular fibrinoid necrosis, was designated as Y or 0 [present; Y, absent; 0].

### Statistical analysis

The data are expressed as mean ± standard error of the mean (SEM), and all the data were analyzed using Prism 10.0 (GraphPad Software, La Jolla, CA, USA), except for the score analysis, which was done using Python v3.58. The data were first analyzed for normality using the Shapiro-Wilk normality test, and after following normality, were analyzed using the analysis of variance (ANOVA), followed by a Bonferroni post-test. For non-parametrical data, it was used the Scheirer-Ray-Hare test followed by the Mann-Whitney U test. The samples were measured more than once, it was corrected for multiple comparisons. The variables analyzed were time, sex, and drug. The significance threshold level was set at 0.05.

## Results

### MAP and HR

**Fig. 1:**
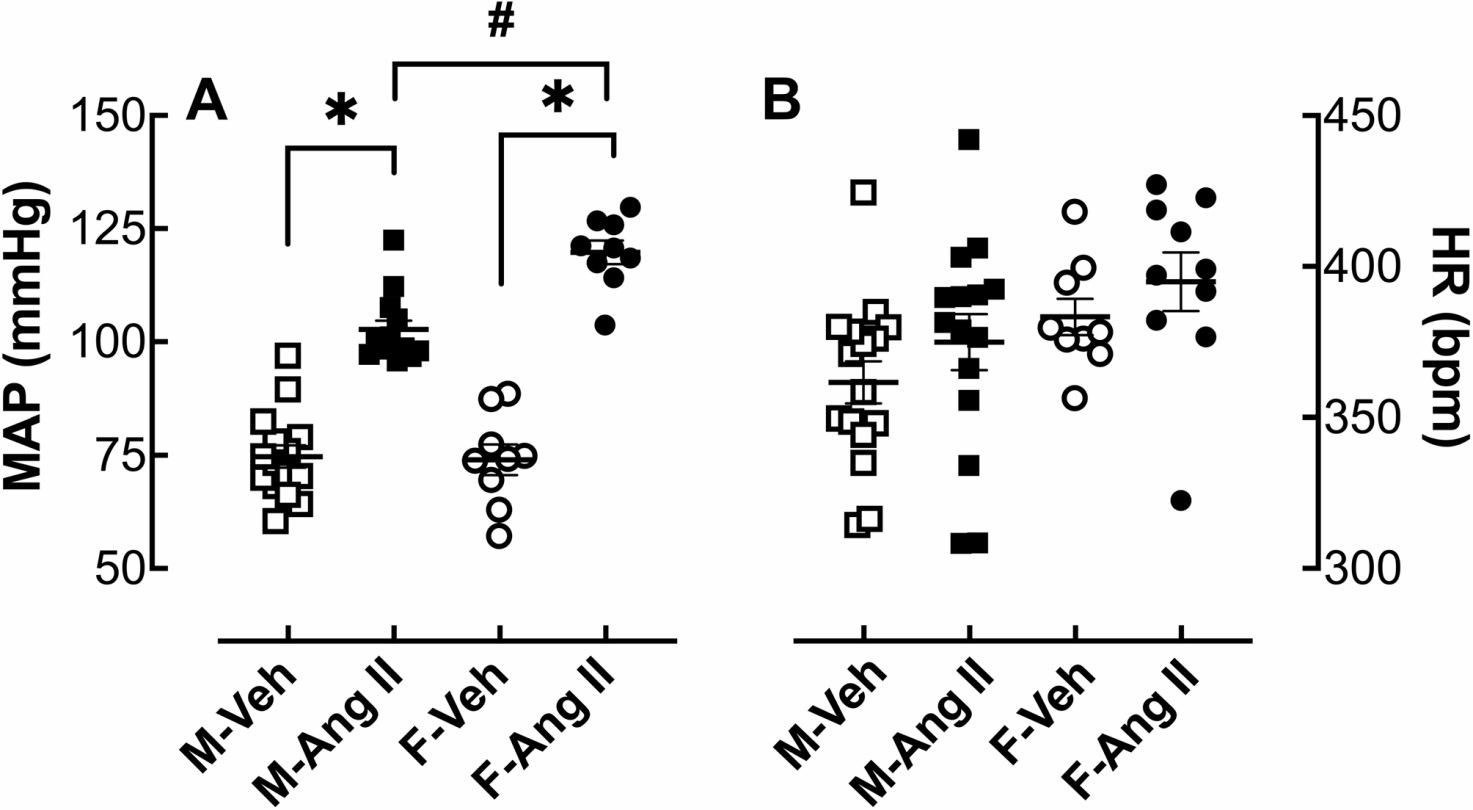
Effect of Ang II infusion on mean arterial pressure (MAP) and heart rate (HR). Male (M) and female (F) hamsters (8 weeks of age) were infused for 4 weeks with vehicle (Veh, open symbol) or 200 ng/kg/min angiotensin II (Ang II; solid symbol) using osmotic minipumps. MAP (**A**) and HR (**B**) were measured at the end of the 4-week infusion period and expressed as the mean ± SEM and were analyzed by two-way ANOVA followed by *Tukey’s post-hoc* test: *p < 0.0001, Ang II vs. Veh; ^#^*p* < 0.0001 M-Ang II vs. M-Ang II, sample size: M-Veh: 15, M-Ang II: 14, F-Veh: 9 and F-Ang II: 9

Four weeks of low-dose (200 ng/kg/min) Ang II infusion increased MAP in male and female hamsters by 27 mmHg and 50 mmHg, respectively (Fig. 1A). Two-way ANOVA showed significant effects of treatment (p^t^< 0.0001) and sex (p^s^<0,005) with an interaction (p^i^<0.002). Ang II infusion had no detectable effect on HR in either sex (Fig. 1B).

### Body weight (BW) and kidney wet weight

**Fig. 2:**
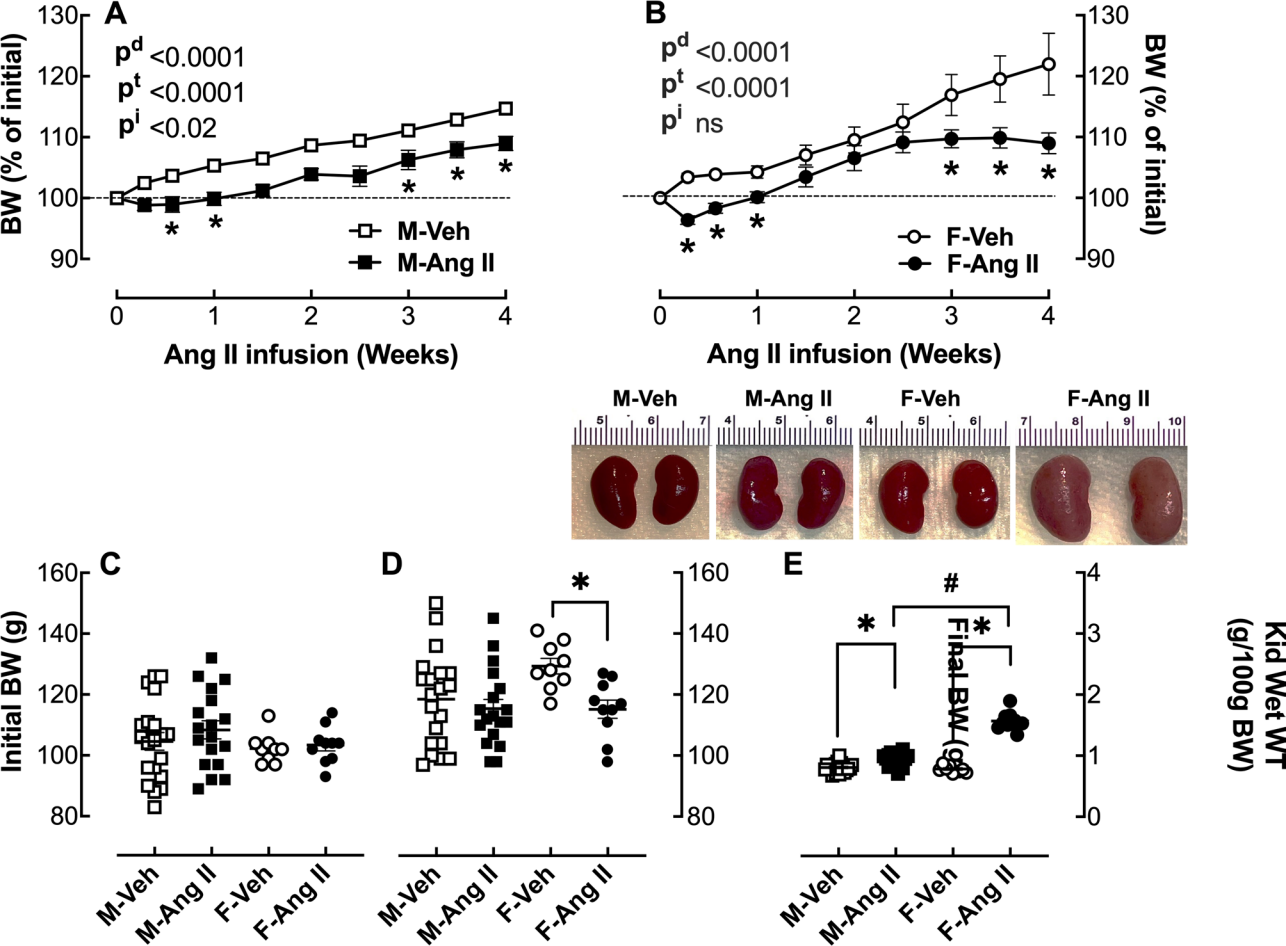
Effect of Ang II infusion on body weight (BW) and kidney wet weight. Male (M) and female (F) hamsters (8 weeks of age) were infused for 4 weeks with vehicle (Veh, open symbol) or 200 ng/kg/min angiotensin II (Ang II; solid symbol) using osmotic minipumps. BW was measured twice weekly during the Ang II infusion period in male (**A**) and female (**B**) hamsters. BW is also expressed as initial (**C**) and final (**D**). Kidney wet weights (WT) were measured and normalized to 100 g BW (**E**), and the kidney morphology is shown as an inset of E. The data are expressed as the mean ± SEM and analyzed by two-way ANOVA (p^d^, effect of drug; p^t^, effect of time; p^i^, interaction) followed by *Tukey’s post-hoc* test: *p < 0.05, Ang II vs. Veh; ^#^*p* < 0.05 F-Ang II vs. M-Ang II, sample size: M-Veh: 20, M-Ang II: 18, F-Veh: 9 and F-Ang II: 10

In both male (Fig. 2A) and female (Fig. 2B) hamsters, Ang II infusion caused an initial drop in BW of 4% and 7% reduction at 2 days, respectively. At 9 weeks of age when the Ang II infusion was initiated, the female hamsters weighed 4.5% less than the males; however, by the end of the four-week infusion period, the female controls weighed 9% more than the males (Fig. 2D). In Ang II-infused females, the weekly BW gain was similar to the vehicle controls up through 16 days, at which point there was no more BW gain for the remainder of the 4-week infusion period, such that Ang-II infused female hamsters weighed significantly (*p* < 0.05) less than vehicle-infused female hamsters, while there was no significant difference in BW between the male and female Ang II-infused hamsters (Fig. 2D).

Ang II infusion slightly increased kidney wet weight in the male hamsters, whereas in females, Ang II significantly increased kidney wet weight by 2-fold (Fig. 2E).

### Food and water intake

**Fig. 3:**
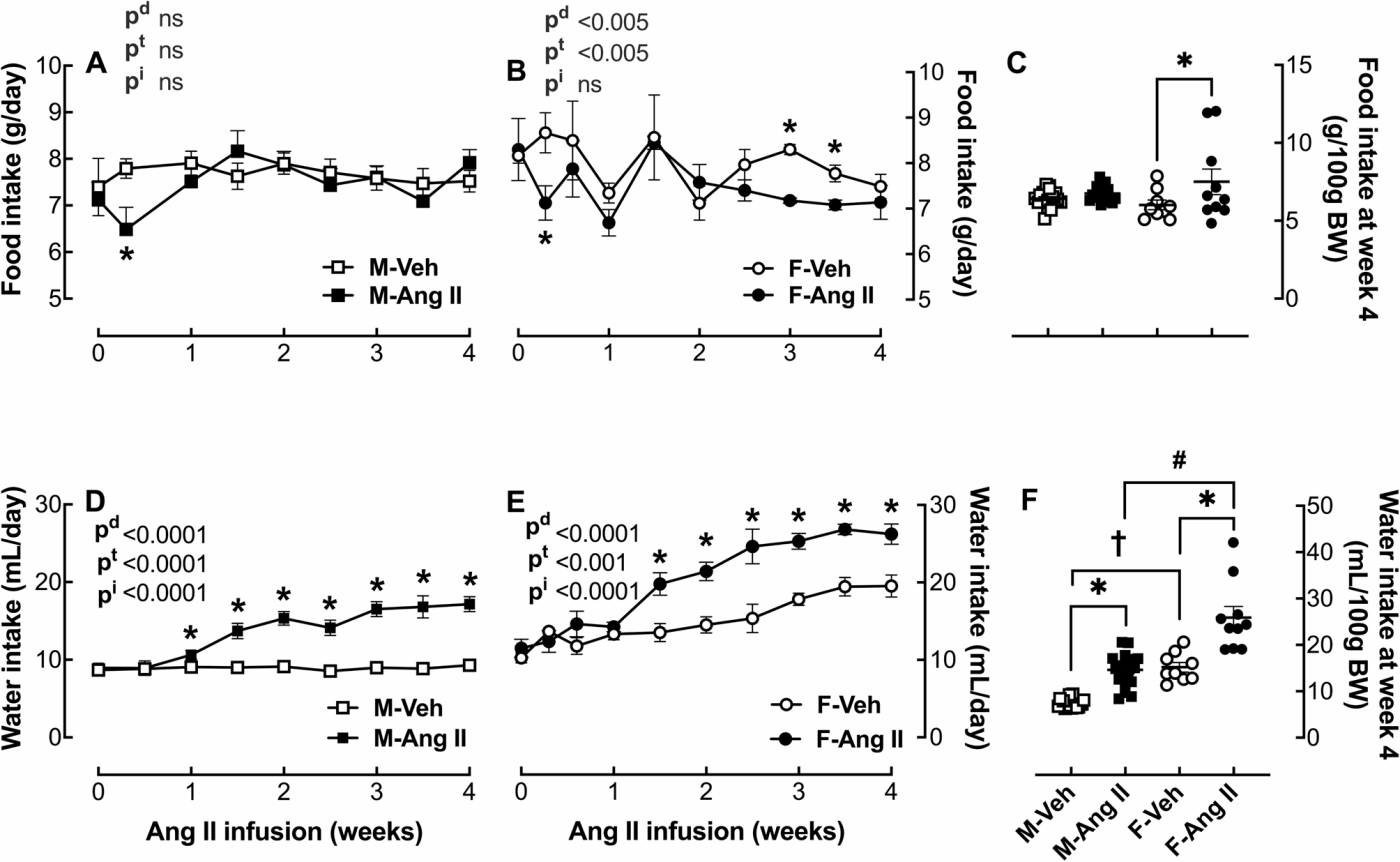
Effect of Ang II infusion on food and water intake. Male (M) and female (F) hamsters (8 weeks of age) were infused for 4 weeks with vehicle (Veh, open symbol) or 200 ng/kg/min angiotensin II (Ang II; solid symbol) using osmotic minipumps. Food (**A** & **B**) and water (**D** & **E**) intake were measured in male (**A**, **D**) and female (**B**, **E**) hamsters during the 4-week infusion period. Final food (**C**) and water (**F**) intake are also expressed and normalized to 100 g BW. The data are expressed as the mean ± SEM and analyzed by two-way ANOVA followed by *Tukey’s post-hoc test* (p^d^, effect of drug; p^t^, effect of time; p^i^, interaction): *p < 0.05, Ang II vs. Veh; ^#^*p* < 0.05, M-Ang II vs. F-Ang II; ^†^*p* < 0.05, M-Veh vs. F-Veh, sample size: M-Veh: 20, M-Ang II: 18, F-Veh: 9 and F-Ang II: 10

In both male (Fig. 3A) and female (Fig. 3B) hamsters, Ang II infusion caused an initial drop in food intake of 9% and 19% within the first week, respectively. Thereafter, in males, the food intake per week was similar to the vehicle-infused animals (Fig. 3A). After the first week, food intake per week was similar between the vehicle and Ang II-infused female hamsters; however, by week 3, food intake declined in the Ang II-infused females for the duration of the experiment (Fig. 3B).

Ang II infusion markedly increased water intake to a similar extent, in both males (Fig. 3D) and females (Fig. 3) throughout the infusion period, though females drank more water than the males regardless of treatment (Fig. 3F).

### Kidney renin-angiotensin system

**Fig. 4:**
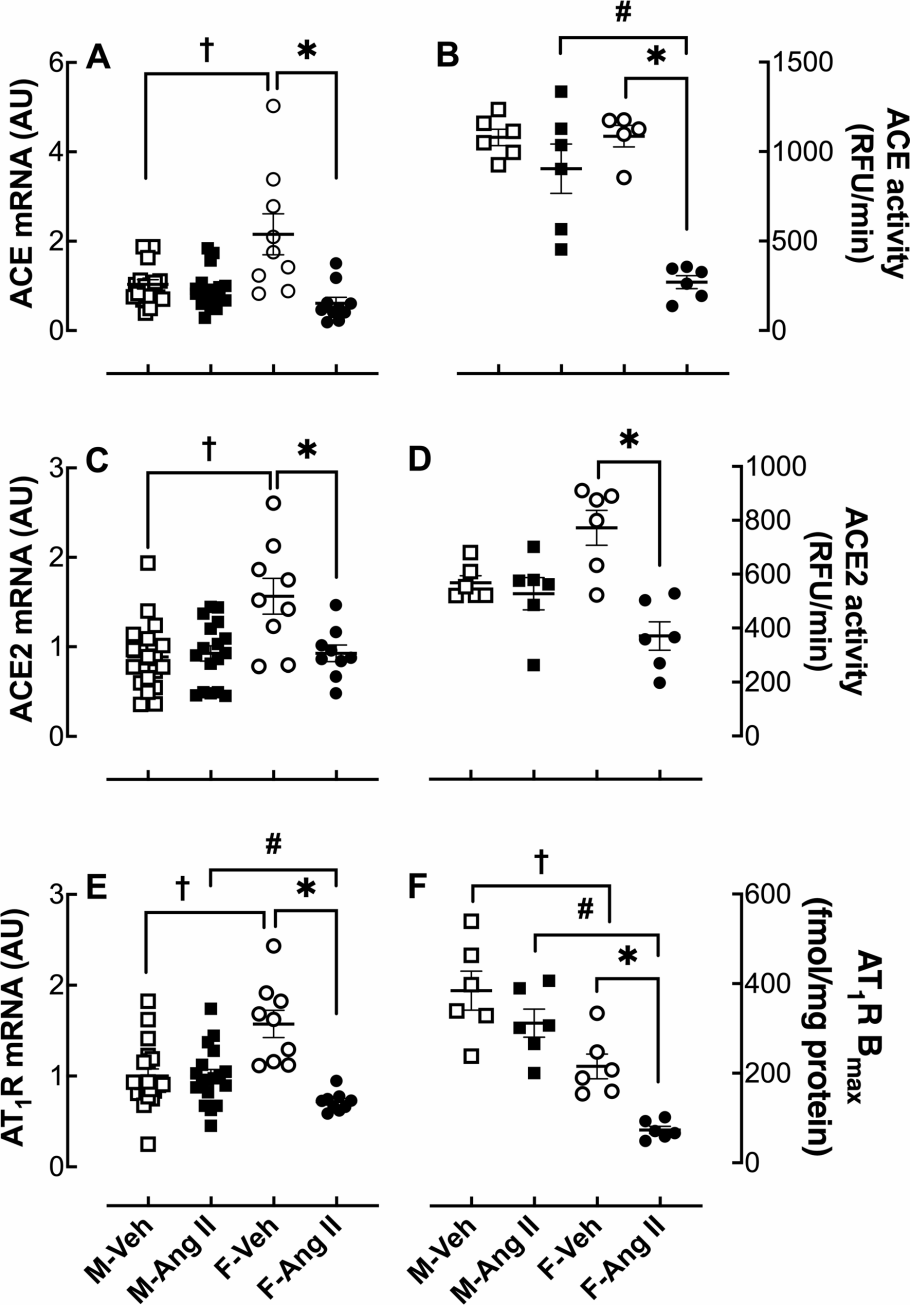
Effect of Ang II infusion on renin renin-angiotensin system. Angiotensin converting enzyme (ACE), angiotensin converting enzyme 2 (ACE2), angiotensin type 1 receptor (AT_1_R) mRNA expression, and ACE/ACE2 enzyme activities were measured in the renal cortex. Male (M) and female (F) hamsters (8 weeks of age) were infused for 4 weeks with vehicle (Veh, open symbol) or 200 ng/kg/min angiotensin II (Ang II; solid symbol) using osmotic minipumps. Renal cortex ACE (**A**) & ACE2 (**C**) mRNA expression, ACE (**B**) & ACE2 (**D**) enzyme activity, AT_1_R mRNA expression (**E**), and renal glomeruli radioligand binding (**F**) were measured at the end of the 4-week infusion period. Data are expressed as the mean ± SEM and were analyzed by two-way ANOVA followed by *Tukey’s post-hoc* test: *p* < 0.05, Ang II vs. Veh; ^#^*p* < 0.05 F-Ang II vs. M-Ang II; ^†^*p* < 0.05, F-Veh vs. M-Veh, sample size: M-Veh: 20, M-Ang II: 18, F-Veh: 9 and F-Ang II: 10 for mRNA and *n* = 6/group for receptor function and enzyme activity

The mRNA expression (Figs. 4A & C) and enzyme activity (Figs. 4B & D) of ACE and ACE2 in the renal cortex were not affected by Ang II infusion in male hamsters. In contrast, Ang II infusion reduced the mRNA expression and enzyme activity of ACE by 72% and 75%, respectively, and ACE2 by 41% and 52% in female hamsters. Moreover, ACE2 mRNA expression (Fig. 4C) and enzyme activity (Fig. 4D) were 40% and 52% lower, respectively, compared to males (*p* < 0.01 and *p* < 0.05, respectively).

The renal cortex mRNA expression (Fig. 4E) and glomeruli radioligand binding (Fig. 4F) of AT_1_Rs were not affected by Ang II infusion in male hamsters. In contrast, Ang II infusion in female hamsters reduced AT_1_R mRNA expression and radioligand binding by 54% and 66%, respectively.

### Renal cytokine expression

**Fig. 5:**
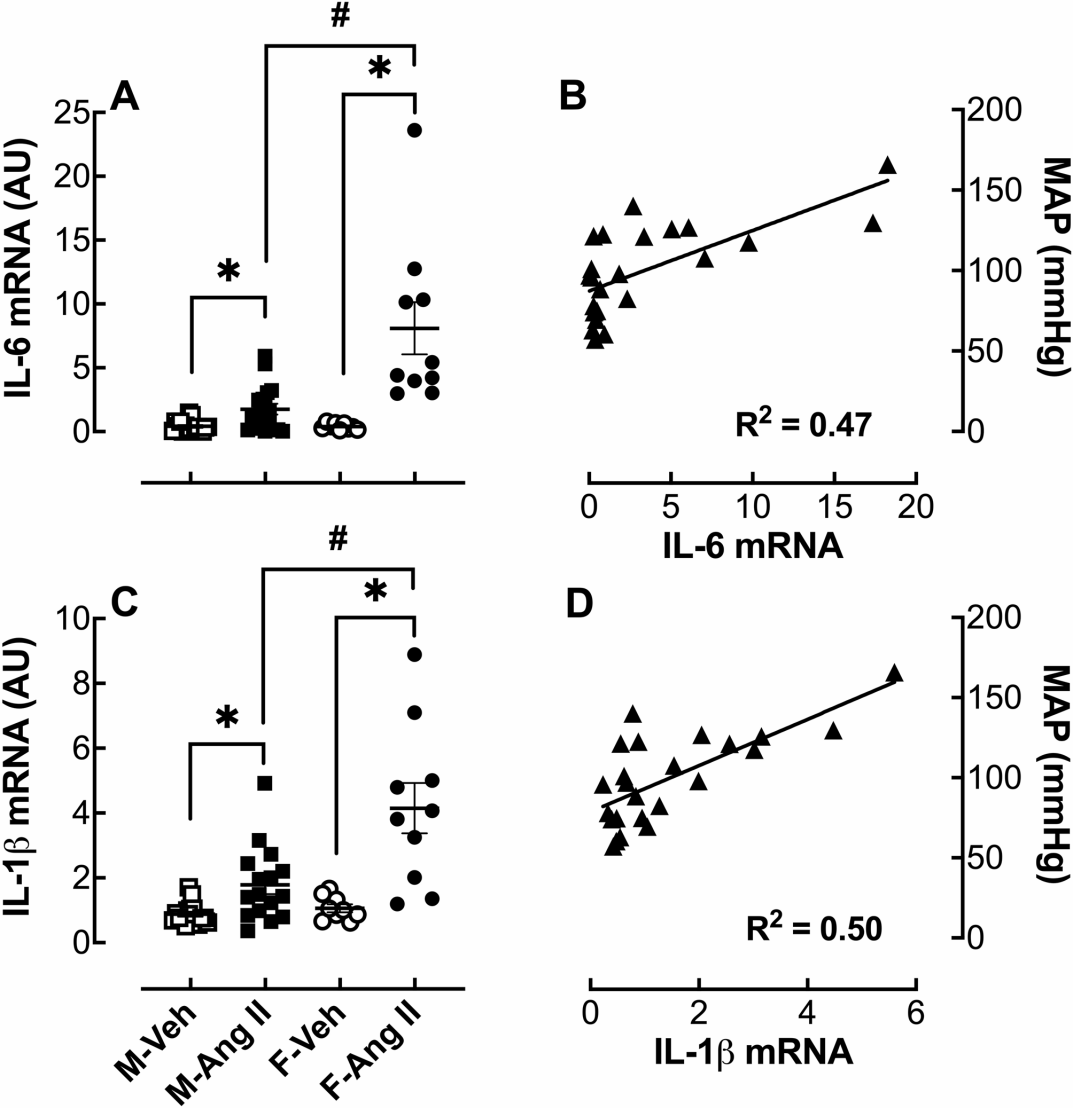
Effect of Ang II infusion on IL-6 and IL-1b mRNA expression in the renal cortex. Male (M) and female (F) hamsters (8 weeks of age) were infused for 4 weeks with vehicle (Veh, open symbol) or 200 ng/kg/min angiotensin II (Ang II; solid symbol) using osmotic minipumps. IL-6 (**A**) and IL-1b (**C**) mRNA expression were measured at the end of the 4-week infusion period. The correlation between mean arterial pressure (MAP) and IL-6 mRNA (**B**) and IL-1b mRNA (**D**) is shown as linear regression curves (solid triangle): *p* < 0.0005 for IL-6 vs. MAP; *p* < 0.0001 for IL-1b vs. MAP. The data are expressed as the mean ± SEM and were analyzed by two-way ANOVA followed by *Tukey’s post-hoc* test: *p* < 0.05, Ang II vs. Veh; ^#^*p* < 0.05 F-Ang II vs. M-Ang II, sample size: M-Veh: 17, M-Ang II: 18, F-Veh: 9 and F-Ang II: 10

There were no sex differences in interleukin (**IL**)−6 (Fig. 5A) and IL-1b (Fig. 5C) mRNA expression in the renal cortex of vehicle-infused hamsters. Ang II infusion moderately increased IL-6 (4.3-fold) and IL-1b (2-fold) mRNA expression in the male hamster renal cortex. In contrast, IL-6 (Fig. 5A) and IL-1b (Fig. 5C) mRNA expression was markedly increased by 20-fold and 3.9-fold, respectively, in the female hamster renal cortex. When the cytokines were correlated with MAP, there was a positive and strong correlation for both IL-6 (R^2^ = 0.4665) and IL-1b (R^2^ = 0.5051) (Fig. 5B & D).

### Renal histology

**Fig. 6:**
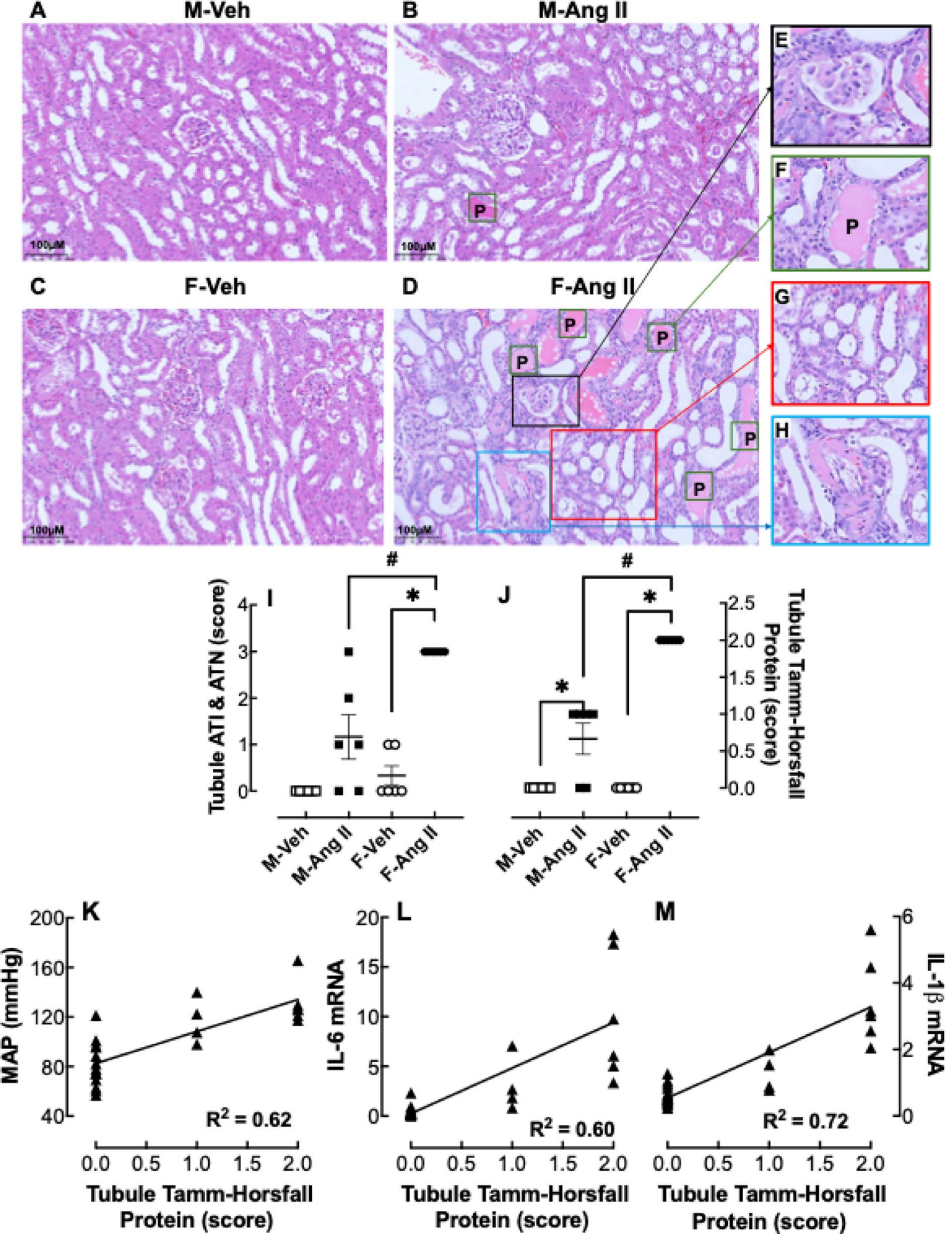
Effect of Ang II infusion on renal histology. Male (M) and female (F) hamsters (8 weeks of age) were infused for 4 weeks with vehicle (Veh, open symbol) or 200 ng/kg/min angiotensin II (Ang II; solid symbol) using osmotic minipumps. Representative images of renal cortex tissue stained with H&E of Veh- (**A**, **C**) and Ang II-infused (**B**, **D**) hamster kidneys are shown. Black box (inset E) is showing glomerular fibrinoid necrosis/ischemic change; Green box (inset F) marked with *P* shows Tamm-Horsfall protein; red box (inset G) shows acute tubular injury/acute tubular necrosis; blue box (inset H) shows vascular fibrinoid necrosis. Twenty-four specimens were examined by two pathologists (D. Kwon and J. Ahn), who were blinded to the group assignment of the experimental hamster kidneys. Quantitation of the degree of acute tubular injury/acute tubular necrosis (ATI & ATN) (**E**) and tubular Tamm-Horsfall Protein (**F**) were graded on a scale of 0–4 [0 (normal);1, affected area up to 1–25% (minimal); 2, affected area 26 − 50% (moderate); 3, affected area 51–75% (moderate-severe); and 4, affected area 76 − 100% (severe)]. The presence of other pathologic findings, including glomerular sclerosis, glomerular congestion, glomerular fibrinoid necrosis/ischemic change, interstitial inflammation, tubular calcification, and vascular fibrinoid necrosis, was designated as Y or 0 [present; Y, absent; 0] (for details see Table 2). The acute tubular injury/acute tubular necrosis (**I**) and tubule Tamm-Horsfall protein (**J**) are expressed as the mean ± SEM and were analyzed by non-parametric two-way ANOVA (Scheirer-Ray-Hare) test followed by Post-hoc pairwise Mann-Whitney U tests: *p* < 0.05, Ang II vs. Veh; ^#^*p* < 0.05 F-Ang II vs. M-Ang II. The correlation between tubule Tamm-Horsfall Protein and MAP (**K**), IL-6 mRNA (**L**), and IL-1b mRNA (**M**) are shown as linear regression curves (solid triangle): *p* < 0.0001, tubule Tamm-Horsfall Protein vs. MAP; *p* < 0.0001, tubule Tamm-Horsfall Protein vs. IL-6; *p* < 0.0001, tubule Tamm-Horsfall Protein vs. IL-6. Sample size: M-Veh: 6, M-Ang II: 6, F-Veh: 6 and F-Ang II: 6

When renal histology in vehicle-infused hamsters (Fig. 6A & C) was compared to Ang II infusion (Fig. 6B & D), there was evidence of renal injury in both sexes; however, the females exhibited more acute tubular injury and necrosis (Fig. 6I) and tubule Tamm-Horsefall protein expression (Fig. 6J) and glomerulus fibrinoid ischemic changes (Fig. 6E) than the males. There was also a positive correlation of tubule Tamm-Horsfall protein with MAP (Fig. 6K), IL-6 (Fig. 6L), and IL-1b (Fig. 6M).

**Table 2:** Renal histological analysis

Histological parameter	M-Veh (*n* = 6)	M-Ang II (*n* = 6)	F-Veh (*n* = 6)	F-Ang II (*n* = 6)	Note
Sclerosis (G)	0	0	0	0	
Congestion (G)	0	0, 0, 0, Y, 0, 0	0	0	
Fibrinoid Necrosis/Ischemic Changes (G)	0	0, 0, 0, Y, Y, Y	0	Y	See Fig. 6E
Inflammation (G)	0	0	0	0	
Calcification (T)	Y, 0, 0, 0, 0, 0	Y, Y, 0, 0, 0, 0	0	Y, Y, 0, 0, Y, Y	
ATI & ATN (T, score)	0	0, 1, 0, 1, 3, 2	1, 0, 0, 0, 1, 0	3 for all	See Fig. 6G and I
Tamm-Horsfall Protein (T, score)	0	0, 1, 0, 1, 1,1	0	2, 2, 2, 2, 2, 2	See Fig. 6F and J
Extravassation of RBC (V)	0	0	0	0	
Fibrinoid Necrosis (V)	0	0, 0, 0, 0, 0, Y	0	Y, Y, Y, Y, Y, 0	See Fig. 6H

Note: G- Glomerulus; T- Tubule; V- Vessel

## Discussion

A major finding of this study is that young female Syrian hamsters have greater pressor responses to Ang II than age-matched males (Fig. 1). These observations differ from reports on other rodent species. Chronic Ang II infusion-induced increases in blood pressure have been shown to be greater in male compared to female C57/BL6 mice [16, 17], and Sprague-Dawley [18] and Spontaneously Hypertensive rats [19] (SHR). Blood pressure studies have been limited in the hamster. Chronic blood pressure measurements by implanted telemetry are difficult in this species because of their thin and inelastic skin. They also tend to bite off the transmitter, and most seriously, have poor survival after transmitter implantation. Data Sciences International, who make implantable radio transmitters, recommends not using hamsters for telemetry studies. We were only successful in recording blood pressure in one out of six hamsters implanted with DSI mouse transmitters. The other five were unsuccessful for the following reasons: one died the day after surgery during anesthesia for carprofen (pain reduce drug) injection; one died 2 days post-surgery without any abnormal discovery during autopsy; one bit off the transmitter a few days later leaving a 2 × 2 cm wound on the skin; one had no signal detection after surgery and it was discovered later that the catheter was no longer in the vessel; and one had their legs paralyzed. These experiences led us to choose terminal blood pressure measurements with a pressure transducer via the femoral artery.

Similarly to our observations of no sex differences in basal MAP in the hamster, Kato et al. [20] reported no detectable sex differences in basal systolic, diastolic, or MAP in a study of 4 male and 7 female hamsters. Jin et al. [21] measured MAP using a two-kidney, one clip (2K1C) technique to mimic renovascular hypertension in the hamster; however, these studies were conducted only in male hamsters. Myers et al. [22] also used the 2K1C technique and only conducted their blood pressure studies in females. Other investigators using the 2K1C model did not always include blood pressure measurements [23, 24], and sometimes the sex of the hamster was missing [25]. Thus, it remains unknown if similar sex differences are apparent in another Ang II-dependent hamster model of hypertension.

Additional evidence that the female hamster is more sensitive to the adverse effects of Ang II is the BW drop during the last two weeks of the 4-week Ang II infusion period (Fig. 2B), which is likely a result of their reduced food intake (Fig. 3B). Ang II infusion at 1000 ng/kg/min was reported to reduce food intake and BW in other rodents including mice [16, 17] and rats [18]^,^ [19] by approximately 6–13% as a secondary effect of increased sympathetic tone, systematic catabolism and reduced appetite. Hamsters are more sensitive to Ang II than mice and rats with higher doses (e.g., 500 ng/kg/min), causing greater drops in BW and increased mortality (*Data not shown*). The infusion dose of Ang II in the 4-week osmotic infusion pump (200 ng/kg/min) is calculated on the initial BW of each hamster. At the beginning of the study, the 9-week-old females weighed 9% less than the age-matched males; however, two weeks later, their BW surpassed the males, and by 13 weeks, at the end of the infusion period, they weighed 10% more than the male hamsters. Thus, the female hamsters received a lower total amount of Ang II when normalized to their final BWs. Ang II is a potent dipsogenic agent in all mammalian species studied, including humans and hamsters [26]. The females drank more water than the male hamsters (Fig. 3D&E); however, there were no sex differences in the magnitude of the Ang II stimulation on drinking behavior (Fig. 3F).

While much is known regarding how Ang II regulates the RAS in mice and rats, less is understood in the hamster. Ang II infusion had no detectable effect on ACE (Fig. 4A&B), ACE2 (Fig. 4C&D), and the AT_1_R (Fig. 4E&F) in the male hamster kidney; however, all three RAS components were down-regulated by Ang II in the female. Similarly to our findings in hamsters, we previously showed in wild-type mice that ACE2 mRNA and enzyme activity are higher in the kidneys of male compared to female wild-type mice [16].

We previously showed Ang II regulation of renal AT_1_Rs is sex specific in the mouse. Similarly to our observations in hamsters, female AT_1_Rs in mouse glomeruli were down-regulated by Ang II infusion under conditions that did not down-regulate the receptor in the males [16]. Taken together, these findings suggest the renal RAS is more tightly regulated in the female than in the male hamster. One important observation was the direct correlation between gene expression and function for the enzyme assays and the absence of correlation for the AT_1_R, similar to what is reported in other species [27–29]. Further investigation into the mechanisms regulating the hamster RAS, including gonadal hormone regulation, would shed light on the role of the RAS in female susceptibility to Ang II-induced pathophysiology in the hamster.

Increased renal expression of IL-6 and IL-1b is associated with hypertension. Elevated renal IL-6 is consistently linked to increased renal injury in both induced and genetic rodent models of hypertension. Reducing IL-6 by genetic knockout attenuated hypertension induced by Ang II infusion and limited renal damage in mice [30]. Neutralizing IL-6 antibodies attenuated the salt-induced increase in MAP in Dahl salt-sensitive rats [31].

IL-1b also significantly contributes to Ang II-induced hypertension and renal injury. Inhibition of IL-1 by administering an anti-IL-1b antibody (01BSUR) in Ang II-infused mice attenuated both the Ang II pressor response and associated renal damage [32]. Genetic knockout of IL-1b also protected mice from Ang II-induced hypertension [33]. Thus, the increase in these two proinflammatory cytokines in the female kidney likely contributed to the greater pressor response to Ang II.

The second major finding of our study is that the female hamster exhibited far more acute tubular necrosis and calcification, glomerular sclerosis, congestion and fibrinoid necrosis and interstitial inflammation in response to Ang II infusion than the male (Fig. 6). This greater degree of renal pathology and inflammation observed in the female renal cortex coincided with significantly higher kidney wet weight (Fig. 2E) and higher levels of mRNA expression of the pro-inflammatory cytokines IL-6 and IL-1b compared to the male (Fig. 5). Studies show collectively that experimental hypertension especially Ang II-dependent disease states, induces severe renal damage characterized by tubular necrosis, vascular fibrinoid necrosis and congestion, glomerular sclerosis and dystrophic calcification in chronic settings [34–36]. The increased wet weight of the female kidney in response to Ang II is a hallmark of hypertensive pathology across various rodent models, including spontaneous, genetic, renovascular, and comorbid disease paradigms [37–39].

Even though the female hamster is able to markedly down-regulate ACE and the renal AT_1_R far more than the male, this negative feedback of the RAS is insufficient to prevent the greater degree of adverse consequences of Ang II infusion on BP and kidney injury. Thus, the activity of the classic RAS is not a likely culprit in the greater female susceptibility to Ang II-induced injury. This is in contrast to other rodent models of Ang II-dependent hypertension, where the ability of the female to down-regulate the classic RAS is associated with greater protection against Ang II-induced pressor effects and end-organ damage [16]. One model in which no sex differences were observed in the Ang II pressor response is Ang II infusion in the presence of cold stress [40]. There is a higher incidence of white coat hypertension in women [41], and hamster studies suggest that cardiovascular and autonomic responses to homotypic stressors are greater in females than males [42]. Thus, it will be worth studying the role of stress in future studies on sex differences in BP in the Ang II infusion model of hypertension.

The hamster has been used as a valuable small rodent model of other disease states involving the RAS. Cardiomyopathy is the most studied disease model in hamsters. The BIO TO-2 inbred strain of Syrian hamsters naturally develops a progressive form of dilated cardiomyopathy, typically manifesting between 3 and 6 months of age, and ultimately leading to heart failure and death and which is attenuated by inhibitors of the vasoconstrictor arm of the RAS [43, 44]. Other hamster models of disease that involve the RAS include nonalcoholic steatohepatitis induced by a high fat/high cholesterol diet [45], abdominal aortic aneurysms [46], myocardial infarction induced by ligating the left coronary artery [47], and pressure overload induced by surgical aortic constriction [48]. However, in all these Ang II-dependent models of disease, males and females have not been directly compared, nor has the kidney been studied.

Hamsters have also been used in structure-function analyses of RAS components by comparing amino acid sequences and protein function across species [49]. Recently, the majority of hamster studies involving the RAS have focused on ACE2 because of this enzyme’s role in serving as the receptor for severe acute respiratory syndrome coronavirus 2 **(**SARS-CoV-2) and because unlike mice and rats, the SARS-CoV-2 virus efficiently replicates in Syrian hamsters causing lung injury and disease features consistent with coronavirus disease 2019 (COVID-19) [50] due to the ability of the hamster ACE2 to bind tightly to the spike protein [51].

Mortality is higher in men with predialysis chronic kidney disease compared to women [52]. Men also start renal replacement therapy earlier than women suggesting that kidney function declines faster in men with chronic kidney disease, although it is not possible to rule out that more women than men choose conservative care over renal replacement therapy. Most animal models of chronic kidney disease show males progress more rapidly than females [52]. However, there are specific renal diseases that are more prevalent and more progressive in women. These include lupus nephritis (nine times more prevalent in women), autoimmune renal vasculitis (women experience more persistent renal involvement), secondary effects on the kidney from interstitial cystitis (90% of patients are women), and pregnancy-related kidney disease. Thus, the female hamster warrants further investigation as a novel model of female vulnerability to hypertension-associated renal pathology and inflammatory disease [9].

In conclusion, the Ang II-infused Syrian hamster is a novel model of female vulnerability to Ang II-dependent disease including hypertension, renal inflammation and fibrosis. Thus, this hamster model of Ang II-induced hypertension complements the wide use of mice and rats as small rodent models to elucidate the role of the RAS in hypertension and associated renal diseases.

### Study limitations

The AT_2_R may play a role in blood pressure regulation in female and male hamsters; however, a limitation of this study is the inability to assess the potential role of AT_2_R, as the hamster AT_2_R has not yet been cloned, and its function has not been characterized in pharmacological studies.

## Data Availability

No datasets were generated or analysed during the current study.
